# The effects of *Allium cepa* L. (onion) and its active constituents on metabolic syndrome: A review

**DOI:** 10.22038/ijbms.2020.46956.10843

**Published:** 2021-01

**Authors:** Amin Galavi, Hossein Hosseinzadeh, Bibi Marjan Razavi

**Affiliations:** 1 School of Pharmacy, Mashhad University of Medical Sciences, Mashhad, Iran; 2 Pharmaceutical Research Center, Pharmaceutical Technology Institute, Mashhad University of Medical Sciences, Mashhad, Iran; 3 Department of Pharmacodynamics and Toxicology, School of Pharmacy, Mashhad University of Medical Sciences, Mashhad, Iran; 4 Targeted Drug Delivery Research Center, Pharmaceutical Technology Institute, Mashhad University of Medical Sciences, Mashhad, Iran

**Keywords:** Allium cepa L., Diabetes mellitus, Dyslipidemia, Hypertension, Metabolic syndrome, Obesity, Onion, Quercetin

## Abstract

Metabolic syndrome as a clustering disorder includes excess abdominal fat distribution, abnormal insulin and glucose metabolism, disturbed blood lipids, pro-inflammatory state, and hypertension. Regarding to the adverse effects of synthetic medicines, the identification of appropriate healthcare approaches, such as herbal medicines, with fewer side effects is more favorable. *Allium cepa* L. (onion) is a culinary and medicinal herb belonging to the family of *Amaryllidaceae*. Flavonoids such as quercetin and kaempferol, alk(en)yl cysteine sulfoxides including S-methyl cysteine sulfoxide and S-propyl cysteine sulfoxide, cycloalliin, thiosulfinates, and sulfides are main compounds existing in the plant. *A. cepa* and its pharmacologically active constituents display broad-spectrum activities including anti-oxidant, anti-inflammatory, lipid-modifying, anti-obesity, antihypertensive, and antidiabetic effects. Our objective in this review was to find out the role of *A. cepa* and its bioactive phytochemicals as cardiovascular protective agents in different metabolic syndrome risk factors, including hyperlipidemia, high blood glucose, obesity, and hypertension.

## Introduction

Metabolic syndrome as a clustering disorder comprises of excess abdominal fat distribution, abnormal insulin and glucose metabolism, disturbed blood lipids, pro-inflammatory state, and hypertension. This syndrome, increases cardiovascular disease (CVD) incidence and mortality by 2-fold and all-cause death by 1.5-fold (1, 2). CVDs account for about 17.8 million annual loss of life all over the world, which makes them the underlying cause of death (3). Based on the modified NCEP (National Cholesterol Education Program) definition, a manifestation of any three or more of five medical conditions presented in Table 1 is required to confirm the clinical diagnosis of metabolic syndrome (4).

Over the last few decades, medicinal herbs are becoming extensively popular among the public and scientific community in both developing and developed countries for being cost-effective, easily accessible, and with relatively lesser adverse effects (5).

*Allium cepa* L. (onion) is a culinary and medicinal herb belonging to the botanical family of *Amaryllidaceae*. This ancient cultivated plant with edible bulbs is the third most important horticultural crop after potato and tomato and widely consumed throughout the world, mainly for its distinctive flavor (6). 

Onion has been utilized during thousands of years for remedial purposes. For instance, it was used by athletes in ancient Greece for purifying their blood and rubbed down by Roman gladiators to firm up the muscles. Hippocrates, the Greek physician, prescribed onion for diuretic effect, healing wounds, and combating pneumonia. It was recommended by medieval doctors to alleviate cough, headache, snake bite, hair loss, and other diseases (6, 7). In traditional medicine, onion has been used for a large variety of ailments such as headache, fever, toothache, cough, sore throat, flu, baldness, epilepsy, rash, jaundice, constipation, flatulence, intestinal worms, low sexual power, rheumatism, body pain and muscle cramps, high blood pressure, and diabetes (8-10).

**Figure 1:**
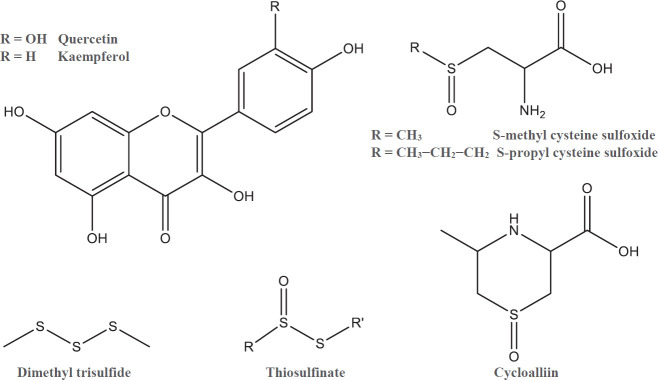
Chemical structures of main flavonoids and sulfur-containing compounds of *Allium cepa*

The results of composition analyses have indicated that *A. cepa* is rich in two valuable chemical groups called flavonoids and alk(en)yl cysteine sulfoxides. The various layers of several varieties of onion bulbs contain quercetin and kaempferol as main flavonoids with anti-oxidant and free radical scavenging benefits ranging from 14.5 to 5110 and 3.2 to 481 µg/g of dry weight, respectively, so that higher amounts are found in red and violet onions and outer layers. S-methyl cysteine sulfoxide (SMCS), S-propyl cysteine sulfoxide, cycloalliin, thiosulfinates, and sulfides are organo-sulfuric compounds existing in the plant (11, 12). Figure 1 illustrates the chemical structures of major onion isolated ingredients. 

Many modern studies have reported that *A. cepa* and its pharmacologically active constituents display broad-spectrum activities, including anti-oxidant (13), anticancer (14, 15), anti-scar (16), hepatoprotective (17), antiplatelet (18), antithrombotic (19), immunoprotective (20), anti-inflammatory (21), anticholelithogenic (22), neuroprotective (23), antibacterial (24), and antifungal (25) properties. They can also be useful in the treatment of allergy (26), asthma (27), hyperuricemia (28), anxiety, depression, and cognitive disorders (29). Onion as a dietary vegetable and its nutraceuticals may assist in cardiovascular risk reduction through anti-oxidant and antithrombotic effects, regulating blood lipids, improvement of endothelial function, and decreasing waistline (30, 31). 

The aqueous onion extract was examined to evaluate its toxicity in the hepatic and pulmonary tissues of rats. Intraperitoneal (IP) administration of onion at a high dose (500 mg/kg/day) induced histologic damages and was lethal to 25% of animals after 4 weeks. However, treatment with low doses (50 mg/kg/day), especially when given orally exhibited no toxic effects (32). In human studies, apart from heartburn, no other adverse effects have been reported following the use of onion (33, 34).

This review aimed to find out the role of *A. cepa* and its bioactive phytochemicals as cardiovascular protective agents in different metabolic syndrome risk factors, including hyperlipidemia, high blood glucose, obesity, and hypertension. For this purpose, we revised the most relevant *in vitro*, animal, and human investigations. Tables 2 and 3 report a summary of selected studies which focused on *A. cepa* and associated components potential efficacy and mechanisms of action in the context of metabolic syndrome and related complications. 

***Methodology***

The literature review was carried out by searching the databases of PubMed, Scopus, and Web of Science using the following key terms: *Allium cepa*, onion, quercetin, metabolic syndrome, dyslipidemia, cholesterol, diabetes, hyperglycemia, insulin resistance, cardiovascular disease, atherosclerosis, hypertension, and obesity. Articles about the effects of *A. cepa* on metabolic disorders written in the English language without publication time restriction have been incorporated in this research. The reference lists of the collected articles were also investigated to recognize further studies. 

***Effects on lipid profile***

Dyslipidemia which is characterized by a combination of four abnormalities in serum lipid profile including elevated levels of total cholesterol (TC), triglyceride (TG), and low-density lipoprotein cholesterol (LDL-C) and low level of high-density lipoprotein cholesterol (HDL-C) raises the incidence of cardiovascular morbidity and mortality (35). Numerous animal and human research projects on the lipid-modifying effect of *A. cepa* have exhibited an important decrease in serum and hepatic values of TG and cholesterol (36).

***In vitro studies***

It has been documented that S-propyl cysteine acts as a reducing agent in the release of apolipoprotein B100 (apoB100), TG, cholesterol, and phospholipids from HepG2 cells. Moreover, the inverse relationship between the rate of apoB100 secretion and carbon chain length in S-propyl, S-ethyl, and S-methyl substitutions of cysteine was detected (37). L-glutamyl-L-phenylalanine (EF) dipeptide, which can be found on average in the range of 2.03 to 4.04 mg per 100 g of fresh onion cultivars, suppressed SREBP-1c (sterol regulatory element-binding protein 1c) and its target genes related to lipogenesis by stimulating AMPK (adenosine monophosphate-activated protein kinase) in AML12 cell line. Consequently, EF caused lower fat accumulation in mouse hepatocytes, depending on the used concentration (38). Quercetin is found to provide prophylaxis of atherosclerosis. This flavonoid stimulated cholesterol removal through the expression of PPAR-γ (peroxisome proliferator-activated receptor gamma) and ABCA1 (ATP-binding cassette transporter A1) in human THP-1 macrophages and thus prevented foam cells formation, which is crucial in atherosclerotic plaque development (39). In order to compare the potency of quercetin, estradiol, and three phytoestrogens (genistein, biochanin A, and daidzein) in protecting LDL from oxidative stress, LDL was isolated from the blood of healthy postmenopausal females not receiving hormonal treatment. After induction of oxidation in LDL, malondialdehyde (MDA) was considered as an indicator of oxidative stress level. Quercetin showed the most antioxidative ability, and its potency was almost 8 times that of estradiol and 100 times that of genistein. In the case of biochanin A and daidzein, the required concentration to reduce 50% of MDA was extremely high. The results of this *in vitro* study also indicated that quercetin, in combination with estradiol, had a synergistic effect (40). 

***Animal studies***

In cholesterol-treated rabbits, the three-month use of onion essential oil (1 g/kg/day) was effective in the reduction of plasma cholesterol and fibrinogen values and aorta lipid content. It also markedly induced the rise of blood clotting time and fibrinolytic activity. The superiority of onion over clofibrate (33 mg/kg) was proved in all parameters mentioned above (41). The blood samples of high cholesterol-fed rats showed a significant decrease in TC, LDL-C, TG, and phospholipids and a rise in HDL-C after 6 weeks treatment with 5% and 10% dehydrated onion powder. Furthermore, the concentration of anti-oxidant compounds such as total thiols, glutathione, α-tocopherol, and ascorbic acid in addition to resistance against peroxidation of lipids increased during the examination (42). 

The cholesterol-lowering effect of onion peel extract was explored in hypercholesterolemic-diet-given mice for 12 weeks. The results revealed that the oral gavage of 100 and 200 mg/kg of the extract, especially the latter one, ameliorated TG and TC contents and weight of liver, cardiac risk factor (TC/HDL-C), atherogenic index ((TC − HDL-C)/HDL-C) as well as plasma TC and LDL-C. The possible mechanisms might be the up-regulation of LDL receptor (LDL-R) and cholesterol 7 alpha-hydroxylase (CYP7A1) expression, and as a result of that, enhanced fecal elimination of cholesterol (43). Red onion extract and quercetin could produce improving effects on paraoxonase 1 (PON1) activity, scavenging of free radicals, LDL oxidation, and lipid peroxidation against oxidative stress induced by HgCl_2_ in rats (44). The prolonged administration of quercetin contributes to regulating utilization of fatty acids in rat lung as the target tissue. Receiving 500 mg/kg/day of the flavonoid for 41 weeks led to a rise in the expression of genes associated with catabolism of fatty acids such as LPL (lipoprotein lipase) and ACOX1 (acyl-coenzyme A oxidase 1). This effect was supported by the reduction of free fatty acids concentration in the serum (45). Adding cycloalliin to the atherogenic diet of rats at doses of 0.1% and 0.3% for 14 days considerably resulted in diminished serum TG levels (about 40%) whereas there were not any significant changes in TC, HDL-C, weight and lipid content of liver, and activities of lipogenic enzymes (46). Another study revealed that SMCS has hypolipidemic activities in Sprague–Dawley rats fed a cholesterol-rich diet which is comparable to gugulipid (50 mg/kg/day) as a natural lipid-lowering drug derived from *Commiphora mukul *tree. SMCS (200 mg/kg/day, by gavage for 45 days) significantly reduced activities of total LPL in the adipose tissue and hepatic malic enzyme, serum and tissue levels of free fatty acids, TG, cholesterol, and phospholipids but significantly increased liver glycogen and fecal excretion of bile acids and neutral sterols when compared to the control group (47). In a recent study, the preventive capacity of *A. cepa* against nonalcoholic fatty liver disease (NAFLD) in the presence of risk factors has been demonstrated. Receiving 7% onion powder significantly reversed the elevation in hepatic tumor necrosis factor alpha (TNF-α) gene expression and plasma levels of alanine transaminase (ALT), aspartate transaminase (AST), glucose, insulin, and TG in fat- and sugar-enriched diet rats; while the increase in consumed food, body weight, gamma-glutamyl transferase, alkaline phosphatase (ALP), cholesterol, and LDL-C did not change significantly after 7 weeks of intervention. The analysis of liver histology showed a remarkable enhancement in lobular and portal inflammation, hepatic steatosis, and ballooning degeneration (48). An earlier investigation showed that onion ingestion alone is not enough for treating NAFLD, but in combination with a healthy dietary pattern has therapeutic potential (49).

***Clinical studies***

Oral daily administration of 100 ml onion juice in volunteers with mild hypercholesterolemia after 8 weeks restrained lipid peroxidation and extended the lag time required for LDL oxidation by reducing oxidative stress. It also decreased the plasma values of TC, LDL-C, and LDL-C/HDL-C ratio by 10.2%, 7%, and 6.8%, respectively, but did not influence TG and HDL-C (*P<*0.05) (50). The results of a randomized controlled clinical trial on women with polycystic ovary syndrome (PCOS) who were 17-37 years old and had body mass index (BMI) 25-40 kg/m^2^ represented the health benefits of onion as a hypocholesterolemic agent. Fifty-four patients were randomly divided into control and therapeutic groups. They received 10-15 g (control group) or 40-60 g (high-onion group) fresh red onion twice a day for 2 months. There was a considerable decline in the serum levels of TC and LDL-C within both groups in comparison to before the intervention. Other lipid markers, including TG, HDL-C, and lipoprotein (a) did not change significantly in any of the groups (33). Even a two-week consumption of *A. cepa* peel extract capsules at a dose equivalent to 100 mg quercetin by normal females aged 20 to 25 years led to a significant reduction in TC, LDL-C, and atherogenic index (34).

Taken together, different mechanisms including the activation of AMPK, decrease in malic enzyme and HMG-CoA reductase activities, up-regulation of LDL-R, increasing expression of CYP7A1 enzyme, lowering cholesterol absorption, enhancing fecal elimination of cholesterol, reduction of lipogenesis and lipid peroxidation, modulating catabolism of fatty acids, and inhibition of apoB100 secretion have been proposed for antihyperlipidemic effect of *A. cepa* and its various preparations. Other studies are necessary to pinpoint the exact mechanisms being responsible for onion effectiveness in dyslipidemia.

***Effects on obesity***

Obesity is a common global health concern. It was estimated that in 2016, the obese population reached over 790 million, and an extra 1.5 billion were overweight. In fact, the prevalence of overweight and obesity combined was nearly 31 percent (51). There is a strong requirement to explore new and efficacious weight-lowering agents because of the limited number of approved safe and efficient drugs, especially for long-term treatment of obesity (52). Research projects have shown the anti-obesity characteristics of onion and phytoconstituents available in this plant such as quercetin and organosulfurs (53, 54).

***In vitro studies***

In a recent study, Funakoshi and colleagues indicated the potential of quercetin in the prevention of adipogenesis in myosatellite cells (MSCs) *in vitro*. This molecule decreased fat cells quantity, TG/protein ratio, and TG amount in cells. Furthermore, the expression of adipogenesis genes such as PPAR-γ and fatty acid binding protein 4 (FABP4) was down-regulated (55). The peel extract of onion (1, 2, 4 μg/ml) and its pharmacologically active component quercetin (1, 2, 4 μg/ml) produced a significant reduction of TG content in 3T3-L1 adipocytes in which effectiveness of the extract was higher than quercetin. Furthermore, glycerol-3-phosphate dehydrogenase (GPDH) activity as an essential enzyme in lipid biosynthesis and mRNA expression of LPL were lowered in this experiment (56). The beneficial effects of quercetin on obesity was demonstrated through down-regulation of C/EBPβ (CCAAT/enhancer-binding protein beta), PPAR-γ, C/EBPα, and FABP4 as adipogenesis-related factors and TG synthetic enzymes such as lipin1, DGAT1 (diacylglycerol acyltransferase 1), and LPAATθ (lysophosphatidic acid acyltransferase). Quercetin also blocked signaling pathways of MAPK (mitogen-activated protein kinase) (ERK [extracellular signal-regulated kinase] and JNK [c-Jun N-terminal kinase]) and mTOR (mammalian target of rapamycin) as well as the release of pro-inflammatory cytokines including monocyte chemoattractant protein-1 (MCP-1), TNF-α, interleukin-1 beta (IL-1β), and IL-6 while stimulated the levels of anti-inflammatory cytokines (IL-10) and anti-inflammatory adipokines (adiponectin) in 3T3-L1 cells (57). In another study, although the low concentrations of quercetin (from 0.5 to 10 µM) were capable of repressing adipogenesis in 3T3-L1 preadipocytes, only the highest concentration (10 µM) appeared as an anti-adipogenic agent in mature fat cells. This effect of quercetin probably results from inducing expression of sirtuin 1. Sirtuin 1 deacetylates proteins and thereby reduces expression and the activity of lipogenic enzyme fatty acid synthase (FAS) (58). 

***Animal studies***

Yoshinari *et al.* evaluated properties of onion extract against body fatness in male Zucker diabetic fatty rats. Four weeks of treatment with the extract (5% w/w) led to a significant decline in the rate of body weight gain, the ratio of liver to body weight, and adipose tissue (mesenteric, pararenal, and epididymal) without altering the satiation. Additionally, onion and a number of its sulfur compounds such as cycloalliin, S-propyl cysteine sulfoxide, S-methyl cysteine, dimethyl trisulfide, and particularly SMCS, could prevent white adipocytes differentiation (59). The addition of quercetin and red onion extract to the high-fat diet significantly lessened inguinal and epididymal adipose tissue weight in mice. Also, the size, density, and morphology of adipocytes altered in varying degrees (60). Quercetin was able to inhibit lipid and TG deposition besides factors associated with the synthesis of TG in cells of zebrafish (57). An oil/water nanoemulsion preparation of quercetin with 33.5-fold greater oral bioavailability compared to free quercetin was used for anti-obesogenic activity in high-fat diet mice. After 10 weeks receiving a dose of 150 mg/kg/day, without a change in the amount of food consumption, body weight gain was prevented by 23.5%. Also, fat mass loss in different regions of the body was found to be 21.2 to 37.4 percent (61). With regard to brown adipocytes role in modulating energy balance, adipose tissue browning seems a promising strategy against obesity (62). In an investigation by Lee *et al.* it was reported that onion skin extract and quercetin have a facilitating role in the transformation of white adipocytes into brown-like adipocytes. This extract (0.5% w/w) caused the up-regulation of various brown adipocyte genes such as PGC-1α (peroxisome proliferator-activated receptor gamma coactivator 1-alpha), PRDM16 (PR domain-containing 16), UCP1 (uncoupling protein 1), CIDEA (cell death-inducing DFFA-like effector A), and FGF21 (fibroblast growth factor 21) in the adipose tissues of mice fed a fat-enriched diet (63).

***Clinical studies***

Onion peel extract capsules, consist of 50 mg quercetin, were administered to Korean overweight and obese participants twice a day for 12 weeks in a double-blind, randomized, placebo-control trial performed in 2013. A decline in BMI and percentage of body fat mass was indicated (64). In a similar study on plasma adipokines, including visfatin, adiponectin, leptin, TNF-α, and IL-4, the only significant change was a rise in adiponectin. However, even this single change occurred in both placebo and intervention groups. It was concluded that inflammatory factors after 12 weeks of supplementation with the extract were unaffected (65).

These studies suggested that several mechanisms such as reduction of fatty acids and TGs biosynthesis, anti-oxidant and anti-inflammatory activity, elevation of adiponectin and energy expenditure, decrease in plasma resistin and insulin resistance, prevention of white adipocytes differentiation, and brown-like remodeling of adipose tissue are involved in anti-obesity properties of onion and its active components. However, further clinical trials are needed to assess the clinical efficacy.

***Effects on hypertension***

Hypertension is the leading metabolic risk factor for the development of life-threatening CVDs, including coronary artery disease, stroke, atrial fibrillation, vascular dementia, and heart failure (66). The decrease of systolic blood pressure as much as 10 mm Hg results in the significant risk reduction in major CVD events by 20%, heart failure by 28%, stroke by 27%, coronary artery disease by 17%, and all-cause mortality by 13% (67). Although the current antihypertensive medications such as angiotensin-converting enzyme (ACE) inhibitors, diuretics, and calcium channel blockers have been proved to be beneficial in blood pressure control, treatment with these medications is often accompanied by undesirable side effects (68). Blood pressure-lowering phytochemicals are gaining growing attention as preventive and curative agents, mainly for prehypertensive patients (69). Several studies have confirmed the antihypertensive potential of onion and quercetin.

***In vitro studies***

The inhibition of ACE by two types of onion (white and purple) aqueous extract was assessed. According to the results, both types were able to reduce enzyme activity in a concentration-dependent way (0–1.25 mg/ml) (70). The binding energy of quercetin with the active site of ACE was calculated −8.5 Kcal/mol in comparison to enalapril as standard (−7.0 Kcal/mol). So, this molecule can reduce blood pressure by inhibiting the conversion of angiotensin I to angiotensin II (71). In a study on rat coronary arteries, it was shown that a physiological concentration of quercetin (10^-7^ moles/l) induces vasodilatation. At least part of this effect is because of the rise in endogenous vasoactive prostanoids in the wall of the coronary arterioles (72). Quercetin is found only in the form of conjugated metabolites in plasma, so, it works through these metabolites. Unlike quercetin, its metabolites do not have direct vasorelaxing action in rat aorta. They might be responsible for *in vivo* improvement of endothelial function (73).

***Animal studies***

The ethanol extract of onion at doses of 0.2, 0.6, 2, and 6 mg/kg was used intravenously in normotensive anesthetized rats. According to the results, onion dose-dependently reduced heart rate and blood pressure. The mechanism of lowering blood pressure can be due to a decrease in heart rate (74). *A. cepa* skin hydroalcoholic extract decreased blood pressure in fructose-fed hypertensive rats presumably by a reduction in oxidative stress and inhibition of Ca^2+^ influx in the cells of vascular smooth muscle (75). The reduction of blood pressure by crude onion was observed in spontaneously hypertensive rats and those with N^ω^-nitro-L-arginine methyl ester (L-NAME)-induced hypertension, probably via anti-oxidant properties (76, 77). The anti-oxidant and antihypertensive activities of onion diminished after 60 min boiling (76). The ethanolic extract of *A. cepa* containing 0.8 mg of quercetin per 100 g body weight did not produce enough blood pressure-lowering effect in spontaneously hypertensive rat (78).

***Clinical studies***

In a randomized controlled study on 22 obese and hypertensive patients, increased systolic and diastolic blood pressure after consumption of a high energy meal was not reduced by the intake of either placebo or 54 mg quercetin (79). The ingestion of 400 mg quercetin increased brachial artery diameter in 15 healthy volunteers depending on dose and time. This flavonoid also induced vasodilation in human arteries *in vitro* (80). In a study on 41 participants with prehypertension and stage 1 hypertension, it was shown that administration of 730 mg/day quercetin for 28 consecutive days significantly lowered blood pressure only in stage 1 hypertensive patients (systolic by 7 ± 2 mm Hg, diastolic by 5 ± 2 mm Hg, and mean arterial pressures by 5 ± 2 mm Hg) while did not have a systemic anti-oxidant influence in both groups (81). The peak effect of receiving an onion macerated in olive oil product equivalent to 2.5 g fresh onion was seen after 5 hrs. At this time, systolic and diastolic blood pressure of 10 subjects with impaired blood fluidity reduced by 10 and 8 mm Hg on average, respectively (82). The incompatible effects of quercetin on blood pressure may be because of apolipoprotein E (APOE) polymorphism. In a double-blind, randomized, placebo-controlled study with a crossover design, 93 overweight or obese adults were treated with 150 mg/day quercetin. The flavonoid controlled blood pressure in APOE3 carriers but was not effective in APOE4 individuals (83).

The above-mentioned investigations have shown hypotensive properties of onion and quercetin which are mediated by several mechanisms such as anti-oxidant activity, ACE inhibitory effect, decreasing heart rate, increasing endothelial nitric oxide, blocking of calcium channels, and vasodilation. More human studies with larger sample size are needed to establish the effects on high blood pressure. In addition, hypotensive properties of other onion phytochemicals would need to be examined in future studies.

***Effects on diabetes and hyperglycemia***

There has been a rising trend in the prevalence of age-standardized diabetes mellitusin adults during 1980-2014. According to estimates, the number of diabetic adults elevated from 108 to 422 million worldwide over this period, and it is expected that it will exceed 700 million in 2025 (84). Diabetes mellitus and its micro-and macrovascular complications, including cardiovascular and kidney diseases, retinopathy, loss of vision, neuropathy, foot ulcers and amputation are important morbidity and mortality causes (85).

***In vitro studies***

Anti-protein glycation of bovine serum albumin with D-fructose and anti-oxidant properties of 25 herbs have been measured. Among them, the skin of *A. cepa* showed the most anti-glycating potential and scavenging capacity of free radicals using 1,1-Diphenyl-2-picrylhydrazyl (DPPH) test with IC_50_= 16.8±5.0 and 4.49±0.59 μg/ml, respectively (86). The inhibitory activity of quercetin and different parts and sizes of onion were seen on porcine pancreatic α-amylase. Furthermore, it was found that outer layers of onions and smaller ones possess more enzymatic activity inhibition (87). Rat intestinal α-glucosidase was inhibited with IC_50_ values 1.27 and 0.15 mg/ml by ethanolic extract of onion skin and quercetin, respectively (88). Sodium-glucose linked transporter (SGLT1) and glucose transporter 2 (GLUT2) play key roles in intestinal glucose absorption. Onion extract inhibited human SGLT1 expressed in Xenopus laevis oocytes in a concentration-dependent manner with a maximum effect of 86% at 1 mg/ml. The inhibition of expressed human GLUT2 in oocytes by 0.25 mg/ml concentration of onion extract was 78%. Among the onion flavonols, the highest levels of SGLT1 and GLUT2 suppression were observed by quercetin-4′-O-glucoside and aglycone quercetin, respectively. Onion extract was able to reduce glucose transport into mouse jejunal intestinal sections competitively and reversibly (89). Glucose transporter 4 (GLUT4), which is regulated by insulin, transports glucose from the blood into fat and muscle cells. An *in vitro* study revealed that the ethanolic extract of *A. cepa* bulbs stimulates glucose uptake through GLUT4 in L6 myotubes in a dose and time reliant pattern. The insulin-like activities of the extract were exerted by increasing phosphorylation of insulin receptor-β, insulin receptor substrate-1, and protein kinase B (Akt) together with the elevation of GLUT4 content and translocation of this protein to the cell surface (90).

***Animal studies***

Several studies have evidenced the health beneficial hypoglycemic action of onion and its functional constituents in animal models (91-95). Thirty mins after consuming sucrose solution (2.0 g/kg) by five-week-old Sprague-Dawley male rats, the ethanolic extract of onion skin and quercetin (both 0.5 g/kg) showed a significant reduction in blood glucose comparable with acarbose (5.0 mg/kg) as an efficient drug for postprandial hyperglycemia. One hr after administration, the lowering blood glucose effect of the extract disappeared but acarbose maintained glycemia near base value for about 2 hrs (88). In another animal study on alloxan diabetic rats which treated by 100, 300 and 600 mg/kg daily doses of aqueous extract of *A. cepa* for 21 days, a decrease in serum levels of glucose, LDL, TG, TC, AST, ALT, and ALP along with an increase in HDL value was reported. The maximum dose of the extract and 2 mg/kg glibenclamide exhibited approximately the same efficacy. The fraction of the plant containing a kaempferol glycoside improved the diabetic condition (96). A meta-analysis was performed in 2008 on antidiabetic activities of onion extract and SMCS in diabetic rats. The findings showed that onion extract and SMCS significantly contributed to the control of blood glucose and body weight (97). Both quercetin and red onion extract as a food supplement in C57BL/6J mice on a high-fat diet for 9 weeks provoked a reduction in methylation of PGC-1α promoter and the augmentation of NT-PGC-1α expression (98). Quercetin (0.2% w/w almost equal to 1000 mg/day in humans according to dose conversion factor provided by FDA (Food and Drug Administration)) was added to the high-fat diet of C57BL/6J male mice for a period of 10 weeks. It reduced hyperglycemia, hyperinsulinemia, creatinine, and inflammatory indicators such as C-reactive protein. Increase in acyl-coenzyme A oxidase 1 (ACOX1) gene expression in the liver was also observed while no significant alterations occurred in energy expenditure, body weight, and lipid profile (99). Because of the low bioavailability of curcumin, its beneficial antidiabetic properties in combination with piperine and quercetin were evaluated on induced diabetes in rats. After 4 weeks of daily oral feeding by this mixture (CPQ) at the dose of 100 mg/kg, amelioration in fasting plasma glucose, glucose tolerance, LDL, HDL, TG, cholesterol, water, food intake and weight loss was observed in comparison with diabetic control and the animals receiving only curcumin. Obtained results were similar to those of 10 mg/kg/day glibenclamide. It was concluded that small amounts of quercetin and piperine in CPQ may exert their effects by lowering the metabolism of curcumin (100). Thiosulfinate (20 and 40 mg/kg) in diabetic rats possibly through non-competitive α-glucosidase inhibition and pancreatic beta cells stimulation showed improvements in postprandial glycemic control, glucose tolerance, and insulin secretion with an effect comparable to 10 mg/kg acarbose (101). S-methyl cysteine (100 mg/kg/day, orally for 60 days) along with a significant decline in blood glucose, insulin plasma concentration, TNF-α, and HOMA-IR (homeostatic model assessment of insulin resistance) showed anti-oxidant properties in rats receiving high-fructose diet. For example, a significant decrease in the serum amount of MDA and an increase in the levels of reduced glutathione (GSH), glutathione peroxidase (GPx), and catalase (CAT) were detected (102).

Some researchers have studied the effects of *A. cepa* and its bioactive constituents on diabetic complications. For example, Gomes *et al.* showed that quercetin could be useful for the management of diabetic nephropathy. This polyphenol (10 mg/kg/day, orally for 28 days) showed improvement in renal function by reducing proteinuria, the plasma concentration of creatinine, uric acid, and urea as well as increasing creatinine clearance in streptozotocin-induced diabetic nephropathy mice. Among them, modifications in creatinine-related factors were significant. Treatment with quercetin also significantly decreased kidney weight/body weight ratio, glomerulosclerosis, and the formation of apoptotic renal cells. They demonstrated that the anti-oxidant behavior of quercetin could lead to renoprotective results (103). The daily IP injection of aqueous onion extract (500 mg/ml/kg) to diabetic rats for 28 days showed a significant lowering effect on elevated serum amount of thromboxane B2 (TXB2). This extract also resulted in reducing the synthesis of TXB2 and aggregation of platelets, which was induced by collagen and arachidonic acid *in vitro* (104). The ethanolic seed extract of *A. cepa* in streptozotocin-induced diabetic Wistar male rats (200, 400 mg/kg/day, orally for 4 weeks) improved reproductive system performance by affecting factors such as seminiferous tubular diameter, luminal diameter, the volume density of lumen together with raising the generation of primary spermatocytes and spermatids (105). In an experiment which was conducted on mice, onion exhibited neuroprotective benefits in the prevention and therapy of diabetic neuropathy. In addition to the amelioration of hyperalgesia and hyperglycemia, levels of thiobarbituric acid reactive substances (TBARS) and serum nitrite decreased, and GSH level increased. According to the results, the neuroprotection impact of onion might be correlated with its anti-oxidant and hypoglycemic effects (106).

***Clinical studies***

High blood glucose can be induced by taking some medications. Jafarpour-Sadegh *et al.* in a randomized, triple-blind, placebo-controlled clinical trial study showed that dietary raw yellow onion at the doses of 100 to 160 g/day based on BMI index for 8 weeks significantly improved doxorubicin-induced insulin resistance and hyperglycemia in patients with breast cancer undergoing chemotherapy (107). In a clinical trial which was performed on 84 patients suffering from type 1 and 2 diabetes mellitus with an average age of 44±3.87 years, the intake of 100 g raw red onion improved oral glucose tolerance and fasting blood sugar after 4 hrs (108). An onion meal in people with lactose intolerance diminished the maximum blood glucose to a greater extent than those who could tolerate lactose (44.2% versus 19.3%, *P<*0.05). It can be due to the decomposition of quercetin glucosides into quercetin in lactose-tolerant subjects and further inhibition of GLUT2 by glucosylated compounds (109).

Overall, *A. cepa* and its active components may be regarded as prophylactic or therapeutic agents against diabetes through different mechanisms including anti-oxidant, α-glucosidase and α-amylase inhibitory effect, up-regulation of adiponectin receptors, reducing insulin resistance and glucose absorption from intestine, elevation in the liver and muscle glycogen content, increasing insulin secretion and phosphorylation of AMPK, insulin-mimetic actions and GLUT4 translocation in skeletal muscles. Moreover, they exhibit benefits in diabetic complications by renal and neural protective effects, enhancing the function of the male reproductive system, and prevention of atherosclerosis. Unfortunately, there is a lack of clinical-based evidence to support improving complications of diabetes by onion.

**Figure 2:**
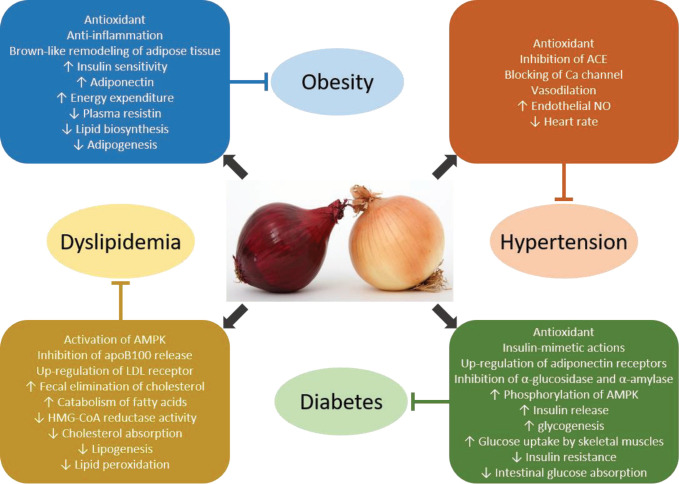
Proposed mechanisms of *Allium cepa* and its main compounds on metabolic syndrome components

Figure 2 shows the mechanisms of action through which onion and onion-derived compounds mediate their biological activities on metabolic syndrome.

**Table 1:** Updated National Cholesterol Education Program (NCEP) criteria for metabolic syndrome (4)

Waist circumference	≥102 cm (men), ≥88 cm (women)
Fasting plasma glucose	≥100 mg/dl or pharmacologic therapy
Serum triglycerides	≥150 mg/dl or pharmacologic therapy
Serum HDL-C	<40 mg/dl (men), <50 mg/dl (women) or pharmacologic therapy
Blood pressure	≥130 mm Hg (systolic) or ≥85 mm Hg (diastolic) or pharmacologic therapy

People with at least three of the above five features are considered to have metabolic syndrome

HDL-C= high-density lipoprotein cholesterol

**Table 2:** A summary of onion and its phytoconstituents effects in metabolic syndrome

**Effect**	**Study design**	**Constituents**	**Results**	**Reference**
**Hypolipidemic**	*In vitro*, human THP-1 cells	Quercetin (0.3 μM)	↑ Protein levels of PPAR-γ, LXRα, and ABCA1, cholesterol efflux to apo-A1 and HDL	(110)
	*In vivo*, male SD rats (received HCD)	Onion high-pressure processing extract (8.1 g/kg/day) for 6 weeks	↑ Fecal total lipid ↓ Food intake, TC, HDL, and LDL Inhibition of HMG-CoA reductase activity	(111)
	*In vivo*, albino rats (atherogenic diet)	Onion extract (equivalent to 2 g/kg of dry onion) for 5 days	↓ Cholesterol, TG, and grade of atherosclerotic lesions	(112)
	*In vivo*, male HCD hamsters	AC powder (5% w/w diet) for 8 weeks	↑ HDL/TC and fecal excretion of neutral and acidic sterols ↓ TC, HDL, non-HDL, non-HDL/HDL, TG, and cholesterol absorption Up-regulation of hepatic SREBP-2, LXRα, and CYP7A1	(113)
	*In vivo*, HFD mice	Quercetin (100 mg/kg) for 8 weeks	↑ SOD, CAT, GSH ↓ Serum TC, TG, and LDL, hepatic cholesterol, TG, and LPO, atherogenic index, AST, ALT, and ALP	(114)
**Anti-obesity**	*In vitro*, 3T3-L1 cells	OPE (100 μg/ml)	↑ CPT-1α and FABP4 mRNA expression ↓ Differentiation of preadipocytes to adipocytes, TG content, and mRNA level of AP2	(115)
	*In vivo*, obesity-induced HFD mice	Red onion extract	↑ Body muscle percentage and energy expenditure ↓ Weight gain and fat mass accumulation Improvement in skeletal muscle mitochondrial number and function	(116)
	*In vivo*, SD rats (received HFD)	OPE (0.36% or 0.72%) for 8 weeks	↑ mRNA levels of CPT-1α and UCP1 in adipose tissues ↓ Retroperitoneal, mesenteric, and total visceral fat, final body weight, and mRNA expression of PPAR-γ, FAS, and ACC in adipose tissues	(115)
	*In vivo*, rats (obesogenic diet)	Combination of quercetin (30 mg/kg/day) and resveratrol (15 mg/kg/day) for 6 weeks	↓ Rate of body weight gain, intra-abdominal and subcutaneous adipose tissue weight Brown-like remodeling of perirenal white adipose tissue	(117)
**Hypotensive**	*In vivo*, high-fructose diet rats	Onion extract (400 mg/kg/day) p.o. for 8 weeks	↑ Heart eNOS activity ↓ SBP, plasma TBARS, aortic NADPH oxidase activity	(118)
	*In vivo*, Wistar HCD rats	Onion powder (100 g/kg diet) for 7 weeks	↑ Erythrocyte SOD and GPx Prevention of impaired endothelium-dependent Ach relaxation in mesenteric arteries	(119)
	*In vivo*, male SD rats with abdominal aortic constriction	Quercetin (estimated 130 mg/kg) for 21 days	↓ Carotid arterial blood pressure, aortic medial thickness, and cardiac hypertrophy	(120)
**Antidiabetic**	*In vitro*	Onion powder and extract	↓ Oxidative stress Inhibition of α-glucosidase activity	(121)
	*In vivo*, AID rats	AC aqueous extract (200, 250, and 300 mg/kg/day) IP for 6 weeks	↓ FBG, total serum lipids, and TC	(122)
	*In vivo*, SID Wistar rats	Onion powder (3% w/w diet) for 8 weeks	↑ Rate of weight gain, plasma albumin ↓ FBG, hepatic cholesterol, TG, and phospholipids, relative liver weight, plasma levels of urea, creatinine, phospholipids, and inorganic phosphorus Improvement in some of diabetic nephropathy clinical parameters	(123)
	*In vivo*, SID rats	Onion essential oil (100 mg/kg) p.o. for 21 days	↑ Insulin, HDL ↓ FBG, TC, TG, LDL, serum NO and TBARS Amelioration of diabetic liver and kidney histology	(124)
	*In vivo*, AID rabbits	AC aqueous extract (300 mg/kg/day) p.o. for one month	↑ Levels of liver CAT, SOD, GPx, and GSH ↓ FBG, liver content of MDA	(125)
	*In vivo*, SID rats fed HFD	OPE (1%) for 8 weeks	↑ Muscle glycogen, liver SOD activity and glycogen, GLUT4 and insulin receptor mRNA expression in skeletal muscle ↓ Liver IL-6 protein and MDA, plasma FFA	(126)
	*In vivo*, male Wistar rats (high-fat-sucrose diet)	Quercetin (30 mg/kg/day) for 6 weeks	↓ Glucose, insulin, HOMA-IR, fructosamine, glucose tolerance test	(127)
	*In vivo*, obese ob/ob mice	Quercetin (30 mg/kg) IP every other day for 10 weeks	↑ GLUT4 protein content in skeletal muscle and insulin sensitivity ↓ FBG	(128)
	*In vivo, *SID rats	Quercetin (1 g/kg diet) for 6 weeks	↓ Water intake, FBG Change in body weight No effect on food consumption	(129)
	*In vivo, *SID Wistar albino rats	Quercetin (15 mg/kg) daily for 4 weeks	↑ TAC, insulin level ↓ Glucose	(130)
	*In vivo, *high fructose-induced diabetic rats	SMC (100 mg/kg/day) for 2 months	↑ HDL and total antioxidant status ↓ FBG, TC, TG, LDL, VLDL, Liver, kidney, and adipose tissue weight, ALT, AST, and body weight	(131)

↑=increase, ↓=decrease, ABCA1=ATP binding cassette transporter A1, AC=Allium cepa, ACC=acetyl-CoA carboxylase, Ach= acetylcholine, AID= alloxan-induced diabetic, ALP= alkaline phosphatase, ALT= alanine transaminase, AP2= activating protein 2, apo-A1= apolipoprotein A1, AST= aspartate transaminase, CAT= catalase, CPT-1α= carnitine palmitoyltransferase 1 alpha, CYP7A1= cholesterol 7α-hydroxylase, eNOS= endothelial nitric oxide synthase, FABP4= fatty acid binding protein 4, FAS= fatty acid synthase, FBG= fasting blood glucose, FFA= free fatty acid, GLUT4= glucose transporter 4, GPx= glutathione peroxidase, GSH= reduced glutathione, HCD= high-cholesterol diet, HDL= high-density lipoprotein, HFD= high-fat diet, HMG-CoA reductase= 3-hydroxy-3-methylglutaryl coenzyme A reductase, HOMA-IR= homeostatic model assessment of insulin resistance, IL-6= interleukin 6, IP= intraperitoneal, LDL= low-density lipoprotein, LPO= lipid peroxidation, LXRα= liver X receptor alpha, MDA= malondialdehyde, NO= nitric oxide, OPE= onion peel extract, p.o.= per os (orally), PPAR-γ= peroxisome proliferator-activated receptor gamma, SBP= systolic blood pressure, SD= Sprague-Dawley, SID= streptozotocin-induced diabetic, SMC= S-methyl-L-cysteine, SOD= superoxide dismutase, SREBP-2= sterol regulatory element binding protein 2, TAC= total anti-oxidant capacity, TBARS= thiobarbituric acid reactive substances, TC= total cholesterol, TG= triglyceride, UCP1= uncoupling protein 1, VLDL= very low-density lipoprotein

**Table 3:** A summary of clinical studies on *Allium cepa* in metabolic syndrome

**Effect**	**Population**	**Constituents**	**Duration**	**Results**	**Reference**
**Hypolipidemic**	24 healthy volunteers with mild hypercholesterolemia	100 mL onion juice	8 weeks	↓ Body weight, BMI, body fat, TC, LDL, and oxidative stress No significant effect on TG and HDL	(50)
	12 normal young Korean women	100 mg quercetin (OPE)	2 weeks	↓ TC, LDL, and atherogenic index	(34)
**Anti-obesity**	72 healthy overweight and obese volunteers	OPE (containing 100 mg quercetin)	12 weeks	↓ Body weight and BMI	(132)
	78 overweight or obese women with PCOS	Quercetin (1000 mg/day)	12 weeks	↓ Weight, BMI, WC, plasma resistin, insulin resistance No significant change in calorie intake	(133)
	70 overweight or obese patients with (pre‑)hypertension	OPE powder capsules (containing 54 mg quercetin) three times a day	6 weeks	No significant differences in serum adiponectin, leptin, and insulin, HOMA-IR, and plasma glucose and TNF-α	(134)
	70 healthy Japanese participants	Onion powder (9 g/day)	12 weeks	↓ Visceral fat only in subjects with lower HDL and ALT No significant effect on abdominal fat, body weight, BMI, AST, and ALP	(135)
**Hypotensive**	70 overweight-to-obese patients with (pre‑)hypertension	OPE powder capsules (contained 54 mg quercetin) three times a day	6 weeks	↓ SBP and MAP	(136)
	92 healthy male smokers	Quercetin capsules (100 mg/day)	10 weeks	↓ SBP and DBP	(137)
**Antidiabetic**	84 PCOS women	Quercetin capsules (500 mg) twice a day	12 weeks	↑ ADIPOR1 and ADIPOR2 transcript expression, AMPK activity ↓ Insulin, HOMA-IR	(138)
	15 healthy young men	3.1 g onion extract	Once 30 mins before the OGTT	Without any significant changes in postprandial blood glucose and insulin and 24 rs urinary glucose output	(89)

↑= increase, ↓= decrease, ADIPOR= adiponectin receptor, ALP= alkaline phosphatase, ALT= alanine transaminase, AMPK= AMP-activated protein kinase, AST= aspartate transaminase, BMI= body mass index, DBP= diastolic blood pressure, HDL= high-density lipoprotein, HOMA-IR= homeostatic model assessment of insulin resistance, LDL= low-density lipoprotein, MAP= mean arterial pressure, OGTT= oral glucose tolerance test, OPE= onion peel extract, PCOS= polycystic ovary syndrome, SBP= systolic blood pressure, TC= total cholesterol, TG= triglyceride, TNF-α= tumor necrosis factor alpha, WC= waist circumference

## Conclusion

The current review article summarized diverse cell, animal, and human studies to realize the role of *A. cepa* and its major ingredients in metabolic syndrome. Based on the gathered data, it has been concluded that onion can be helpful in the prevention and treatment of dyslipidemia, high blood pressure, diabetes mellitus, and obesity as metabolic syndrome disorders and subsequently CVDs on account of anti-oxidant, anti-inflammatory, and vasodilatory properties, increasing insulin release and response, reduction of cholesterol and glucose absorption, modulating the metabolism of lipids and carbohydrates, and inhibition of adipogenesis. These effects are chiefly attributed to the ability of controlling associated signaling pathways, transcription factors, genes expression, enzymes and receptors activities. Additional clinical investigations with sufficient human population and duration are needed to be accomplished to prove the effectiveness and safety of *A. cepa*, especially in the case of potential interaction with conventional drugs.
